# The Association between Vitamin D Deficiency and Diabetic Retinopathy in Type 2 Diabetes: A Meta-Analysis of Observational Studies

**DOI:** 10.3390/nu9030307

**Published:** 2017-03-20

**Authors:** Bang-An Luo, Fan Gao, Lu-Lu Qin

**Affiliations:** 1 Department of Mental Health, Brain Hospital of Hunan Province, Changsha 410007, Hunan, China; luo276@126.com; 2 Department of Social Medicine and Health Management, Xiangya School of Public Health, Central South University, Changsha 410078, Hunan, China; gfydsl@163.com; 3 Department of Prevention Medicine, Medical School, Hunan University of Chinese Medicine, Changsha 410208, Hunan, China

**Keywords:** vitamin D, diabetic retinopathy, type 2 diabetes, meta-analysis

## Abstract

Emerging evidence from in vivo and in vitro studies have shown that vitamin D may play an important role in the development of diabetic retinopathy (DR), but individually published studies showed inconclusive results. The aim of this study was to quantitatively summarize the association between vitamin D and the risk of diabetic retinopathy. We conducted a systematic literature search of Pubmed, Medline, and EMBASE updated in September 2016 with the following keywords: “vitamin D” or “cholecalciferol” or “25-hydroxyvitamin D” or “25(OH)D” in combination with “diabetic retinopathy” or “DR”. Fifteen observational studies involving 17,664 subjects were included. In this meta-analysis, type 2 diabetes patients with vitamin D deficiency (serum 25(OH)D levels <20 ng/mL) experienced a significantly increased risk of DR (odds ratio (OR) = 2.03, 95% confidence intervals (CI): 1.07, 3.86), and an obvious decrease of 1.7 ng/mL (95% CI: −2.72, −0.66) in serum vitamin D was demonstrated in the patients with diabetic retinopathy. Sensitivity analysis showed that exclusion of any single study did not materially alter the overall combined effect. In conclusion, the evidence from this meta-analysis indicates an association between vitamin D deficiency and an increased risk of diabetic retinopathy in type 2 diabetes patients.

## 1. Introduction

Diabetes mellitus (DM) is a large public health problem which affects more than 300 million individuals in the world, with significant morbidity and mortality worldwide [1]. In addition to the deleterious effects of the disease itself, its long-term complications can conspicuously decrease the quality of life of diabetes patients. Diabetes patients with uncontrolled or poorly-controlled blood glucose are at high risk of microvascular complications. Diabetic retinopathy (DR) is among the most common diabetic complications, and is the leading cause of blindness among working-aged individuals worldwide [2]. The prevalence of DR varies from 20% to 80% in different studies. Recent estimates suggest that the number of people with diabetic retinopathy will increase to 191 million by 2030 [3]. Diabetic retinopathy has a complex process. Many risk factors for DR have been established, such as poor glycemic control, long duration of diabetes, smoking, inflammation, obesity, and hypertension. Stratton et al. have given evidence that poor glycemic control and long duration of diabetes are independent risk factors of DR [4]. Praidou et al. found that increased physical activity is associated with less severe levels of DR, independent of the effects of HbA1c and body mass index (BMI) [5]. However, detailed pathophysiological mechanisms and other DR risk factors are not fully clarified.

Vitamin D is a multi-functional fat-solute metabolite required for humans’ growth and development. Vitamin D deficiency (VDD) is seen across all ages, races, and geographic regions. Due to the wide functionality of vitamin D and because vitamin D deficiency is epidemic, vitamin D’s non-classical functions are gaining more attention for the close association between vitamin D deficiency and cancers, infectious diseases, autoimmune diseases, diabetes, and diabetic complications [6,7,8]. The prevalence of vitamin D deficiency is high in type 2 diabetes mellitus (T2DM) patients [9,10,11]. Vitamin D receptors are expressed extensively in the retina [12], and an animal study showed that calcitriol was a potent inhibitor of retinal neovascularization in an oxygen-induced ischemic retinopathy mouse model [13]. This evidence indicated that vitamin D may play a role in the pathogensis of diabetic retinopathy.

While there are accumulating studies on the effect of vitamin D on diabetic retinopathy, the association between vitamin D and diabetic retinopathy are conflicting. According to some studies, vitamin D deficiency is associated with an increasing risk of diabetic retinopathy. Patrick et al. found an association between serum 25-hydroxyvitamin D concentration and diabetic retinopathy in a cohort of 1790 type 2 diabetes patients [14]. Inukai et al. reported that serum 25-hydroxyvitamin D (25(OH)D) levels were decreased in type 2 patients with retinopathy when compared with type 2 patients who had no microangiopathy [15]. However, others suggested that no significant differences in vitamin D status were found between type 2 diabetes with or without diabetic retinopathy. Alam et al. found no association between serum 25(OH)D levels and the presence and the severity of diabetic retinopathy [16].

Currently, there is insufficient evidence showing whether serum vitamin D deficiency is related to diabetic retinopathy, and the determination of this relationship has rarely been conducted. To address these issues, we carried out this meta-analysis by pooling the results from observation studies to examine the potential association between vitamin D and diabetic retinopathy.

## 2. Methods

### 2.1. Data Sources

Relevant articles were identified by a systematic literature search of Pubmed, Medline, and EMBASE through September 2016, using the following keywords: “vitamin D” or “cholecalciferol” or “25-hydroxyvitamin D” or “25(OH)D” in combination with “diabetic retinopathy” or “retinopathy” or “DR”. Additionally, we manually searched all eligible original articles, reviews, and other relevant articles. This meta-analysis was performed following the guidelines for observation study protocols (Meta-analysis Of Observational Studies in Epidemiology (MOOSE) guidelines) [17].

### 2.2. Study Selection

Original articles evaluating the relationship between serum vitamin D status and diabetic retinopathy were reviewed and selected if they met the following inclusion criteria: (a) the study population was type 2 diabetes patients; (b) DR was the outcome, and the control group consisted of type 2 diabetes patients without DR; (c) the study presented sample sizes and odds ratios (OR) with 95% confidence intervals (CI) or information that could be used to infer these results; (d) vitamin D deficiency was defined as a 25(OH)D level below 20 ng/mL, and vitamin D insufficiency was defined as 25(OH)D levels of 21–29 ng/mL; (e) the study was published in English; and (f) the study met the predefined methodological quality assessment criteria for observational studies (Supplementary Data Box 1). Animal experiments, chemistry, or cell-line studies and editorials, commentaries, review articles, and case reports were excluded. Other exclusion criteria consisted of studies with a score of 0 for any item or a total score <7 out of 10 maximal points [18].

Two independent reviewers (Bang-An Luo and Fan Gao) reviewed all the literature searches and acquired full-length articles for all citations meeting the predefined selection criteria. Final inclusion or exclusion decisions were made after reading the full text. We resolved any disagreements through consensus or arbitration by a third reviewer (Lu-Lu Qin).

The following information was extracted from each article: the last name of first author, publication year, location of study, study design, sample size, 25(OH)D assay methods, 25(OH)D concentration, and the prevalence of vitamin D deficiency. In the case of relevant missing data, contacts were made to the main authors for more information.

### 2.3. Statistical Analysis

We performed this meta-analysis with RevMan Software (Version 5.2, Cochrane Collaboration, London, UK). The odds ratio (OR) and weight mean difference (WMD) were used as measures of associations between vitamin D status and risk for DR. If the OR and 95% CI were not available for the meta-analysis, these data were extracted from the selected articles to construct 2 × 2 tables of serum low vitamin D status versus the presence or absence of diabetic retinopathy.

We used forest plots to visually assess pooled estimates and corresponding 95% CIs for each study. The heterogeneity among the results of the included studies was evaluated with *I*^2^ statistical tests. The percentage of *I*^2^ < 25, near 50, and >75 indicated low, moderate, and high heterogeneity, respectively. A fixed-effects model or a random-effects model was used to combine the study results according to the heterogeneity. Once the effects were found to be heterogeneous (*I*^2^ > 50%), a random effects model was used. Otherwise, a fixed effect model was used.

Potential publication bias was assessed using the funnel plots. A sensitivity analysis was conducted to test the robustness of results, as well as to investigate the effect of a single article on the overall risk estimated by removing one article in each turn. Additionally, a subgroup analysis was performed to explore the possible explanations for heterogeneity. All the *p-*values were for a two-tailed and *p* < 0.05 was considered as statistically significant.

## 3. Results

### 3.1. Search Results

**Figure 1:**
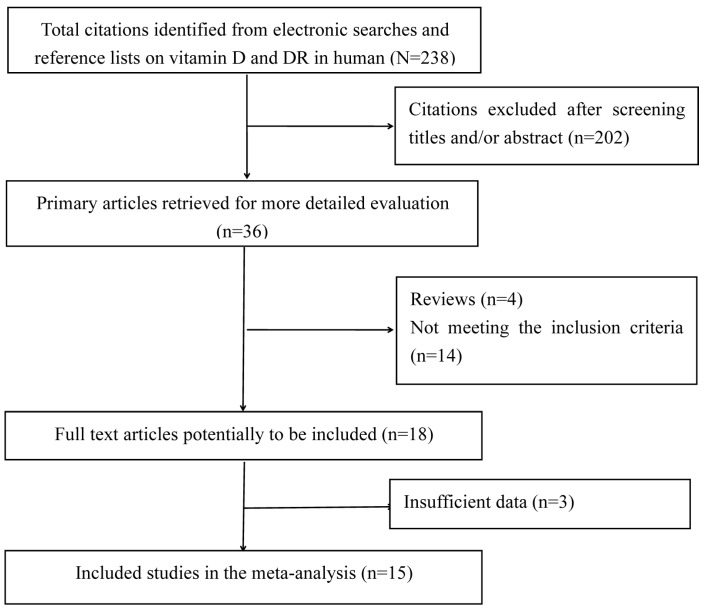
Flow chart of literature search and study selection.

Of the 238 articles identified from our initial search, a total of 15 studies were finally identified as eligible for inclusion in this meta-analysis through a strict screening process (Figure 1). The quality assessment showed that the quality scores of these studies ranged from 8.5 to 10 according to the MOOSE guidelines, which indicates that all of the selected studies were of high quality (Supplementary Table S1).

### 3.2. Characteristics of the Included Studies

The characteristics of these articles in this meta-analysis are summarized in Table 1. These studies were published from 2000 to 2016. Among these studies, nine were conducted in Asia, two each in North America and Europe, one in Africa, and one each in three countries (Australia, New Zealand, and Finland). Seven studies had cross-sectional design, five were case–control, two were prospective cohort, and one had a retrospective cohort design. Four different assay techniques were used to measure the serum 25(OH)D levels, and four different ways were used to diagnose DR. Eight of the included studies explored the association between vitamin D deficiency and diabetic retinopathy, four explored the association between vitamin D insufficiency and diabetic retinopathy, and ten explored the mean difference in vitamin D status among diabetic retinopathy and non-diabetic retinopathy.

The diversity of participant characteristics was considerable in these studies. Out of 17,664 participants, 3455 (19.6%) were diagnosed with diabetic retinopathy, consisting of different ages and races. In addition, the age of type 2 diabetes participants were older than 18 years and the mean BMI—if provided by articles—varied from 13.9 to 42.0 kg/m^2^. The vitamin D status of diabetic retinopathy ranged from 9.2 to 32.6 ng/mL.

### 3.3. Main Analysis

**Figure 2:**
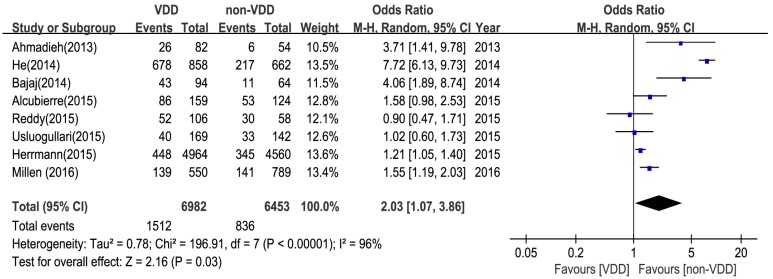
The meta-analysis of the association between vitamin D deficiency (VDD) and diabetic retinopathy (DR).

The relationship between serum vitamin D deficiency (VDD) and the risk of diabetic retinopathy is shown in Figure 2 and Supplementary Table S2. Eight studies involving 13,435 participants were included. As the results of this meta-analysis show, type 2 diabetes with vitamin D deficiency (serum 25(OH)D levels <20 ng/mL) had an increased risk of developing diabetic retinopathy (OR = 2.03, 95% CI: 1.07, 3.86) in the random-effects model.

Based on four studies, the pooled OR for vitamin D insufficiency (VDI, serum 25(OH)D levels <30 ng/mL) was calculated as (OR = 0.89, 95% CI: 0.20, 4.02) (Supplementary Table S2 and Supplementary Figure S1). Only the result of vitamin D deficiency showed that low serum vitamin D status increased the risk of diabetic retinopathy.

**Figure 3:**
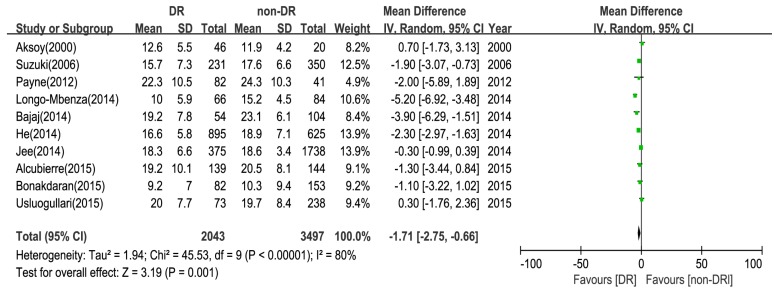
Meta-analysis of the association between 25-hydroxyvitamin D (25(OH)D) levels and DR.

The comparison of the mean difference between the DR group and the control group (non-DR group) is shown in Figure 3. From the results of the random-effects model, the pooled effect was −1.7 ng/mL (95% CI: −2.75, −0.66) and significant heterogeneity was observed (I^2^ = 80%, *p* < 0.001). It showed that serum 25(OH)D levels were significantly lower in diabetic retinopathy patients than the control in type 2 diabetes. This result demonstrated that vitamin D deficiency is significantly related with an increased risk of DR.

### 3.4. Sensitivity and Subgroup Analysis

To explore the impact of various exclusion criteria on the overall risk estimate, we conducted sensitivity and subgroup analyses to examine the potential sources of heterogeneity in this meta-analysis. In the result of the pooled ORs, the study conducted in China [24] was responsible for most of the heterogeneity in this meta-analysis. After excluding this study, the heterogeneity was down to 67% and the pooled OR was 1.50 (95% CI: 1.41, 1.98). Besides, there were no obvious changes in the pooled ORs as a result of the exclusion of any other single study. In the comparison of the mean difference between the DR group and the control group, moderate heterogeneity (*I*^2^ = 70%, *p* < 0.001) was observed among the remaining studies after the exclusion Jee’s study [25]. Jee’s study was responsible for most of the heterogeneity. Further exclusion of any single study did not alter the overall combined relative risk, with a range from −2.2 ng/mL (−3.28, −1.17) to −1.6 ng/mL (−2.43, −0.69), and each outcome had statistical significance.

In the subgroup with study design, there were moderate heterogeneities in cross-sectional and prospective cohort studies for the pooled OR (*I*^2^ = 52%, *p* = 0.15 for cross-sectional studies; *I*^2^ = 61%, *p* = 0.11 for prospective cohort studies) (Supplementary Materials Figure S2). We found that there were obvious heterogeneities in the cross-sectional and the case–control studies for the pooled WMD (*I*^2^ = 79%, *p* = 0.0007 for cross-sectional studies; *I*^2^ = 76%, *p* = 0.006 for case–control studies) (Supplementary Materials Figure S3). In the subgroup with VD assay methods, there were high heterogeneities in the chemiluminescence (CL) vitamin D assay method for the pooled OR (*I*^2^ = 99%, *p* < 0.0001), and the study conducted in China [24] was responsible for the heterogeneities. After excluding this study, the heterogeneity in the CL subgroup was down to 8% and the pooled OR was 1.25 (95% CI: 1.06, 1.48) (Supplementary Materials Figure S4). In the subgroup with DR diagnosis, there were modern heterogeneities in the ophthalmologists group (OR = 1.71, 95% CI: 1.11, 2.62, *I*^2^ = 74%, *p* = 0.0004) (Supplementary Materials Figure S5). Subgroup analyses of BMI, season, duration of diabetes, race, and age were not conducted due to insufficient data in some studies.

### 3.5. Publication Bias

No obvious publication bias was observed in the funnel plots of this meta-analysis (Supplementary Materials Figure S6).

## 4. Discussion

Vitamin D’s non-classical functions have attracted much public health attention for its closer association with some diseases. This is the first meta-analysis of the relationship between serum vitamin D status and DR in type 2 diabetes. In this meta-analysis, the results of 15 observational studies provided strong evidence that serum 25(OH)D levels were associated with an increased risk of DR in type 2 diabetes patients. Both the results of the pooled OR for vitamin D deficiency (serum 25(OH)D levels <20 ng/mL) and the pooled effect of WMD showed that the serum 25(OH)D levels in type 2 diabetes had a relationship with DR, while the pooled OR for vitamin D insufficiency (serum 25(OH)D levels <30 ng/mL) did not. Considering the results of vitamin D deficiency and that serum 25(OH)D levels were more reliable and accurate than the result of vitamin D insufficiency (only four studies), we concluded that low 25(OH)D levels were associated with an increased risk of DR.

The quality of the studies included in this meta-analysis was high. From the funnel plots, we concluded that there was no obvious publication bias in this meta-analysis. We further conducted sensitivity and subgroup analyses to explore potential sources of heterogeneity. From the forest plots, we observed high heterogeneity among the pooled OR and WMD as the association of DR (Figure 2 and Figure 3), which was not surprising given the different characteristics of participants and adjustments for confounding factors. Regarding the results of sensitivity analyses, two studies probably contributed to the heterogeneity [24,25]. After excluding the two studies, there was moderate heterogeneity, and there may be other factors contributing to the heterogeneity in this study. Firstly, there was no consensus on the levels of vitamin D denoting deficiency and insufficiency, and the diagnosis standards of DR might not be uniform among different ophthalmologists in these studies. Secondly, there were four methods for measuring vitamin D, such as HPLC (high performance liquid chromatography) and RIA (radioimmunoassay). Thirdly, the time of collecting blood sample were different. Fourthly, the heterogeneity might be a result of differences in the study populations—there were several races included in this meta-analysis. Fifthly, the duration of diabetes, the age of patients, the diet, the treatment of diabetes, and the sunlight exposure are confounding factors in this meta-analysis. Hence, more studies are needed to give full proof.

There is an epidemic of vitamin D deficiency around the world [6]. From this meta-analysis, the prevalence of vitamin D deficiency was high among type 2 diabetes patients, although it may vary from different latitude, ethnicity, body mass index, season, and supplementation of vitamin D. VDD was consistent with previous studies [9,10,11]. For patients with diabetes, the association with vitamin D deficiency and risk of developing diabetes has been acknowledged. Vitamin D deficiency is associated with an increased risk for diabetes [9,22,29].

In patients with diabetic retinopathy, the level of serum 25(OH)D was lower than patients without diabetic retinopathy. The characteristic feature of diabetic retinopathy is the appearance of vascular lesions of increasing severity, ending up in the growth of new vessels (neovascularization). Vitamin D has anti-inflammation properties and inhibits vascular smooth muscle cell growth and effects on the expression of transforming growth factor β1 [13,33,34,35]. Vitamin D is an important regulator of hundreds of genes regulating key biological processes from cell division to apoptosis [36]. It is well known that poor glycemic control is a risk factor for the development and progression of DR, and vitamin D deficiency has been shown to impair insulin synthesis and secretion in animal models of diabetes [37]. On the other hand, an optimal concentration of vitamin D is strongly proven to be necessary for efficient insulin secretion and function [38,39,40], and vitamin D receptors (VDR) are ubiquitously expressed in every human tissue, including retina. Active vitamin D mediates its biological function by binding to vitamin D receptors. Vitamin D receptors have been found to be associated with insulin secretion and sensitivity, and have been identified in pancreatic beta cells [41]. Additionally, some genes associated with the development of diabetic retinopathy have been found, such as *Bsm1*, *rs2228570*, and *TT*. So, vitamin D status is related with the development and progression of diabetic retinopathy among type 2 diabetes patients.

Increasing studies have given more evidence of this. Annwelier et al. found that the serum 25(OH)D levels were associated with optic chiasm volume [42], and in a vitro experiment, vitamin D was found to inhibit neovascularization in retinal tissue in a model of ischemic retinopathy [13]. What is more, proof of the VDR polymorphisms related with diabetic retinopathy was found. Hong et al. showed that patients with the B allele (BB or Bb) of Bsm1 polymorphism in VDR were associated with lower risk of diabetic retinopathy compared to patients without the B allele (bb) in Korean type 2 diabetic patients [41]. Bucan et al. showed that the bb genotype in VDR has a higher risk of developing diabetic retinopathy [43]. Zhong et al. found that rs2228570 was associated with increased risk of diabetic retinopathy in Han Chinese type 2 diabetes patients [44]. Benjamin et al. reported that the anticipation of retinopathy onset is significantly associated with the exaggeration of oxidative stress biomarkers or decrease of antioxidants in African type 2 diabetics, and supplementation with vitamin D should be recommended as complement therapies of T2DM [26]. Therefore, low serum 25(OH)D levels are association with an increased risk of diabetic retinopathy, but more studies are required to identify the mechanism of the relationship between vitamin D and diabetic retinopathy fully.

It is well known that one of the two major sources of vitamin D is cutaneous synthesis by solar ultraviolet B radiation and the other is dietary intake. The cutaneous synthesis of vitamin D is affected by many factors, such as season, latitude, time of day, skin pigmentation, the amount of skin exposed, and whether makeup with sunscreen is used, so serum 25-hydroxyvitamin D levels vary between areas and persons. Hence, dietary vitamin D supplementation and physical activity might be a feasible way for patients with diabetes to maintain sufficient 25-hydroxyvitamin D levels. Physical activity may increase blood 25-hydroxyvitamin D concentrations as a consequence of an associated increase in sunlight exposure. Praidou et al. reported that increased physical activity is associated with less severe levels of diabetic retinopathy, independent of the effects of HbA1c and BMI [5]. Lee et al. showed that 3 months of vitamin D supplementation improved neuropathic symptoms by 50% in diabetic patients whose 25-hydroxyvitamin D status was deficient at baseline [45]. However, there has yet to be a dietary vitamin D supplementation trial on diabetic retinopathy. Thus, large randomized controlled trials researching on reducing diabetic retinopathy in type 2 diabetes are needed to accurately evaluate the potential benefits of these low-cost interventions in the future.

Diabetic retinopathy may develop and progress to advanced stages without producing any immediate symptoms to the patient. Screening for DR is essential in order to establish early treatment of sight-threatening retinopathy and has been demonstrated to be successful at achieving vision loss [46]. Considering the heavy burden of DR and the association between serum 25(OH)D level and diabetic retinopathy, low 25(OH)D levels may help us to find more early stage diabetic retinopathy patients. Therefore, screening low 25(OH)D levels may be a potential simple way for screening diabetic retinopathy among type 2 diabetes in primary hospitals—especially where there is a shortage of ophthalmic equipment or ophthalmologists.

The main strength of this study is giving strong evidence of the association of serum vitamin D and diabetic retinopathy. However, there were several limitations in this study. Firstly, different diagnostic criteria of diabetic retinopathy and different measured assays could have influenced the pooled effect. Secondly, there were some confounding factors in this meta-analysis, such as age, race, diet, the treatment of diabetes, sun exposure, physical activity, and so on. Some, but not all, of the studies generated adjusted OR, so we could not pool the findings by adjusting for confounding factors. Thirdly, there was a lack of access to complete data of all related published papers, despite correspondence with the authors. Fourthly, the association between vitamin D and the severity of diabetic retinopathy was not analyzed in this meta-analysis because of a lack of enough data and information. Finally, only published articles in English were included.

## 5. Conclusions

In conclusion, this meta-analysis indicates an association between vitamin D deficient type 2 diabetes mellitus patients and an increased risk of diabetic retinopathy. Type 2 diabetes patients with vitamin D deficiency experienced an increased risk of diabetic retinopathy. However, further studies are required to better understand the relationship between vitamin D deficient type 2 diabetes patients and diabetic retinopathy, and well-designed randomized controlled trials are needed to determine the explicit effect of vitamin D supplementation on the prevention of diabetic retinopathy. Considering the high prevalence of vitamin D deficiency and the burden of the diabetic retinopathy, screening type 2 diabetes patients who are at risk of vitamin D deficiency should be considered.

## Figures and Tables

**Table 1:** Characteristics of observational studies included in this meta-analysis.

Author and Year	Country	Study Design	Sample Size (*n*)	VD Assay Method	DR Diagnosis	VDD Prevalence (%)		Mean 25(OH)D ng/mL (SD)		Significant	Adjustment
						DR NDR		DR NDR			
Aksoy (2000) [19]	Turkey	Cross-sectional	66	RIA	Ophthalmologists	NA	NA	12.6 ± 5.5	11.9 ± 4.2	Yes	No
Suzuki (2006) [20]	Japan	Case–control	581	RIA	Ophthalmologists	NA	NA	15.7 ± 7.3	17.6 ± 6.6	Yes	Age, BMI, duration, HbA1c, treatment
Payne (2012) [21]	US	Cross-sectional	123	CL	Ophthalmologists	NA	NA	22.3 ± 10.5	24.3 ± 10.3	Yes	Multivitamin use
Ahmadieh (2013) [22]	Lebanon	Cross-sectional	136	RIA	Ophthalmologists	78.8	53.8	NA	NA	Yes	BMI, duration, smoking
Bajaj (2014) [23]	Indian	Case–control	158	NA	Ophthalmologists	79.6	49.0	NA	NA	Yes	No
He (2014) [24]	China	Cross-sectional	1520	CL	The International Clinical DR Severity Scale	75.7	63.6	16.6 ± 5.8	18.9 ± 7.1	Yes	Age, sex, duration
Jee (2014) [25]	Korea	Cross-sectional	2113	RIA	The Early Treatment Diabetic Retinopathy Study severity scale	NA	NA	18.3 ± 6.6	18.7 ± 3.4	Yes	Sex
Longo-Mbenza (2014) [26]	Congo	Case–control	150	HPLC	The modified Airlie House classification system	NA	NA	10 ± 5.9	15.2 ± 4.5	Yes	No
Alcubierre (2015) [27]	Spain	Case–control	283	CL	Ophthalmologists	61.9	50.7	19.2 ± 10.1	20.5 ± 8.1	Yes	Race, season, physical activity
Bonakdaran (2015) [10]	Iran	Cross-sectional	235	RIA	Ophthalmologists	NA	NA	9.2 ± 7.0	10.3 ± 9.4	No	Age, sex, duration, BMI, HbA1c,BMI, sex, HbA1c
Herrmann (2015) [28]	Australia, New Zealand, and Finland	Prospective	9524	CL	Ophthalmologists	56.5	51.7	NA	NA	Yes	Age, sex, et al. *
Reddy (2015) [29]	Indian	Case–control	164	HPLC	The modified Airlie House classification system	27.0	23.0	NA	NA	No	Duration
Usluogullari (2015) [30]	Turkey	Retrospective	557	HPLC	Ophthalmologists	45.2	54.2	20.0 ± 7.7	19.7 ± 8.4	No	Age, BMI, sex, HbA1c
Zoppini (2015) [31]	Italy	Cross-sectional	715	CL	Ophthalmologists	NA	NA	NA	NA	Yes	Age
Millen (2016) [32]	US	Prospective	1339	LC-MS	The modified Airlie House classification system	49.6	38.8	NA	NA	Yes	Race, duration, HbA1c, hypertension

DR: diabetic retinopathy; VD: vitamin D; VDD: vitamin D deficiency; NDR: no diabetic retinopathy; NA: not available. CL: chemiluminescence; HPLC: high performance liquid chromatography; RIA: radioimmunoassay; LC-MS: high-sensitivity mass spectrometry; SD: standard deviation; et al. *: diabetes duration, HbA 1c, systolic blood pressure, BMI, lipids (triglycerides and high-density lipoprotein (HDL) and low-density lipoprotein (LDL) cholesterol), smoking, baseline use of oral hypoglycemic agents, and baseline use of insulin.

## Supplementary Materials

The following are available online at http://www.mdpi.com/2072-6643/9/3/307/s1, Table S1. Quality scores of included studies on vitamin D status and pregnancy outcomes. Table S2. Results of meta-analysis according to 25-hydroxyvitamin D (25(OH)D) level. DR: diabetic retinopathy; NDR: no diabetic retinopathy. Figure S1. Meta-analysis of the association between vitamin D insufficiency (<30 ng/mL) and DR. Figure S2. Subgroup analysis of pooled ORs according to study design. Figure S3. Subgroup analysis of weight mean difference (WMD) according to study design. Figure S4. Subgroup analysis of pooled ORs according to vitamin D assay method. Figure S5. Subgroup analysis of pooled ORs according to different DR diagnosis. Figure S6. Result of funnel plots.
