# Supplementary material for: The Association between Vitamin D Deficiency and Diabetic Retinopathy in Type 2 Diabetes: A Meta-Analysis of Observational Studies
Source: Nutrients. 2017 Mar 20;9(3):307. doi: 10.3390/nu9030307 (PMC5372970; doi:10.3390/nu9030307)
Supplement: Supplementary file 1 [file nutrients-09-00307-s001.doc]

Supplementary Information: The Association between Vitamin D Deficiency and Diabetic Retinopathy in Type 2 Diabetes: A Meta-Analysis of Observational Studies

Bang-An Luo, Fan Gao and Lu-Lu Qin

**Box 1.** Quality assessment of observational studies (total 10 points) *.

**1. Selection of participants (1/0)**

*Cohort studies (1/0)*

Selected cohort was representative of the general population (population-based studies) or target catchment population (hospital-based studies) (1)

Cohort was a selected unrepresentative group (0)

*Case–control studies (1/0)*

Cases and controls drawn from the same population (1)

Cases and controls drawn from different sources or the selection of groups (0)

**2. Comparability of groups (2/0)**

No significant differences between the groups reported in terms of age, BMI, the duration of diabetes, pre-existing medical conditions were explicitly reported, or these differences were adjusted for (2)

Differences between groups were not examined (1)

Groups differed and no adjustment results provided (0)

**3. Definition of outcomes (2/0)**

Definition of outcomes

Referenced or standard definition (2)

Explicit non-standard definition (1)

Unspecified or unacceptable definition (0)

**4. Ascertainment of outcomes (2/0)**

How the diagnosis was made

Prospectively diagnosed or review of notes/hospital discharge records (2)

Retrospective chart review or database coding (1)

Process not described (0)

**5. Sample size (1/0)**

≥200 participants in a cohort study; ≥50 participants in either group (case/control) (1)

100 ≤ participants < 200 in a cohort; 25 ≤ participants < 50 in either group (case/control) (0.5)

Participants <100 or total number of events <10 in a cohort; participants <25 in either group (case/control) (0)

**6. Study design (2/0)**

Prospective cohort or nested case–control within a prospective cohort (2)

Cross-sectional, case–control, or retrospective cohort (1)

Not described or poorly designed (0)

**Exclusion:** score zero in any item (1 to 6) or a total score <7 out of 10 maximal points

* A score based quality assessment criteria for non-randomized observational studies adapted from Duckitt & Harrington

**Table S1.** Quality scores of included studies on vitamin D status and pregnancy outcomes.

| **Study** | **Selection of Participants** | **Comparability of Groups** | **Outcomes Definition** | **Ascertainment** | **Sample Size** | **Study Design** | **Total Score** |
| --- | --- | --- | --- | --- | --- | --- | --- |
| Aksoy (2000) | 1 | 2 | 2 | 2 | 0.5 | 1 | 8.5 |
| Suzuki (2006) | 1 | 1 | 2 | 2 | 1 | 1 | 8 |
| Payne (2012) | 1 | 2 | 2 | 2 | 1 | 1 | 9 |
| Ahmadieh (2013) | 1 | 2 | 2 | 2 | 1 | 1 | 9 |
| Bajaj (2014) | 1 | 1 | 2 | 2 | 1 | 1 | 8 |
| He (2014) | 1 | 2 | 2 | 2 | 1 | 1 | 9 |
| Jee (2014) | 1 | 2 | 2 | 2 | 1 | 1 | 9 |
| Longo-Mbenza (2014) | 1 | 2 | 2 | 2 | 1 | 1 | 9 |
| Alcubierre (2015) | 1 | 2 | 2 | 2 | 1 | 1 | 9 |
| Bonakdaran (2015) | 1 | 2 | 2 | 2 | 1 | 1 | 9 |
| Herrmann (2015) | 1 | 2 | 2 | 2 | 1 | 2 | 10 |
| Reddy (2015) | 1 | 2 | 2 | 2 | 1 | 1 | 9 |
| Usluogullari (2015) | 1 | 1 | 2 | 2 | 1 | 1 | 8 |
| Zoppini (2015) | 1 | 1 | 2 | 2 | 1 | 1 | 8 |
| Millen (2016) | 1 | 1 | 2 | 2 | 1 | 2 | 9 |

**Table S2.** Results of meta-analysis according to 25-hydroxyvitamin D (25(OH)D) level. DR: diabetic retinopathy; NDR: no diabetic retinopathy.

| **Vitamin D Status** | **25(OH)D ng/ml** | **DR (n)** | **NDR (n)** | **OR** | **95% CI** | **I^2^ (%)** | ***p*** |
| --- | --- | --- | --- | --- | --- | --- | --- |
| Deficiency | <20 | 2348 | 11087 | 2.03 | 1.07,3.86 | 96 | 0.000 |
| Insufficiency | <30 | 1351 | 1796 | 0.89 | 0.20,4.02 | 98 | 0.000 |


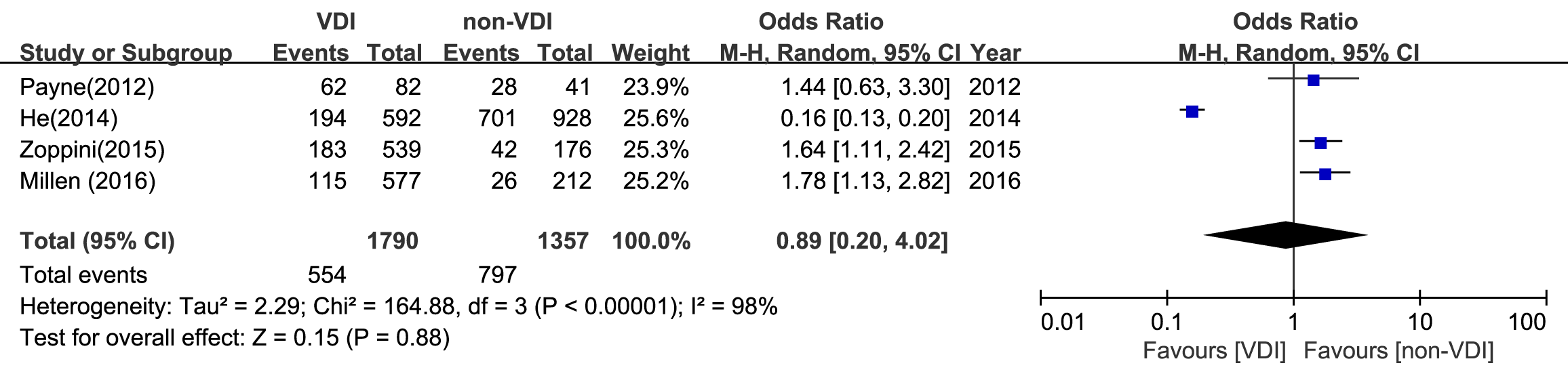


**Figure S1.** Meta-analysis of the association between vitamin D insufficiency (<30 ng/ml) and DR.

**
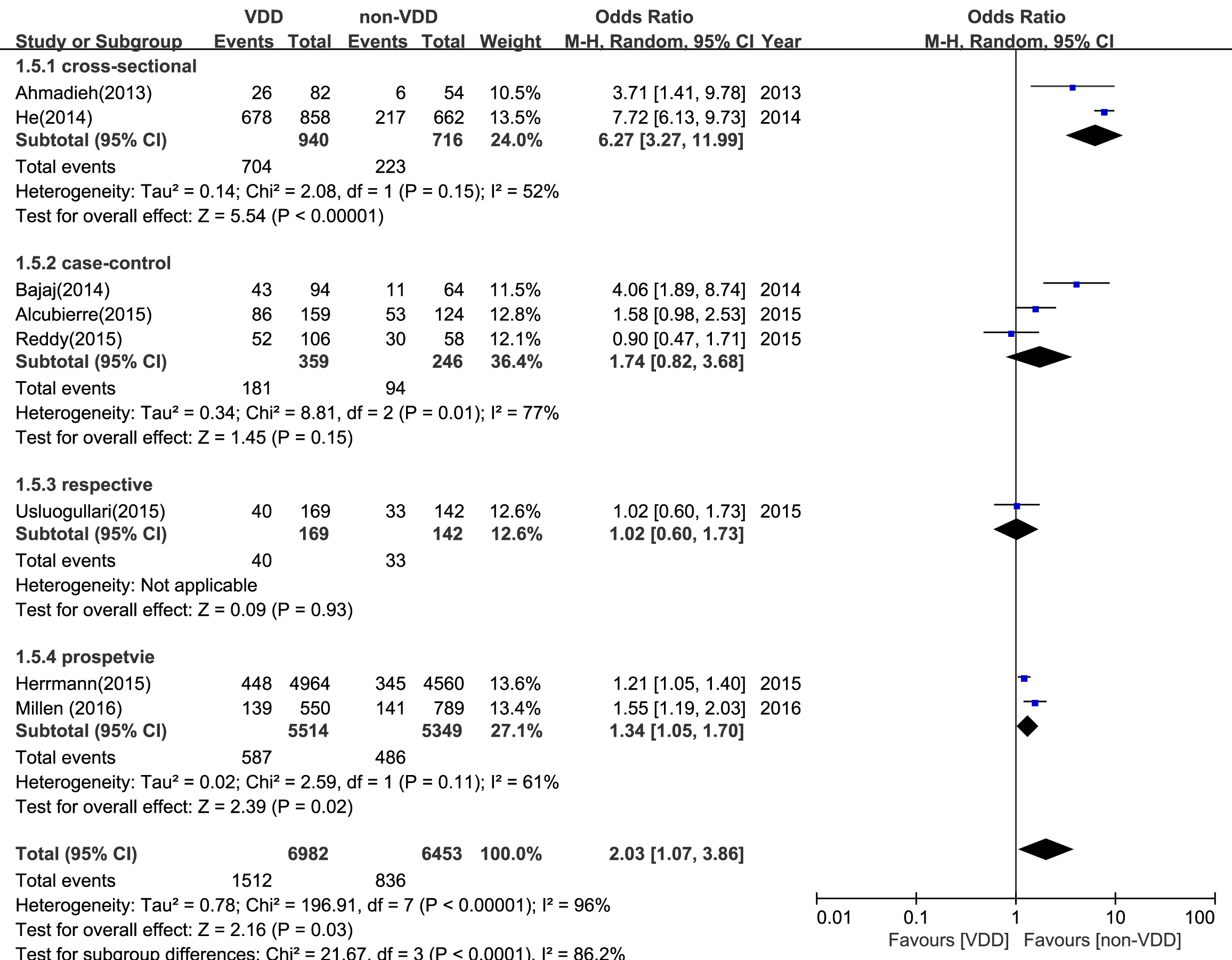
**

**Figure S2.** Subgroup analysis of pooled ORs according to study design.


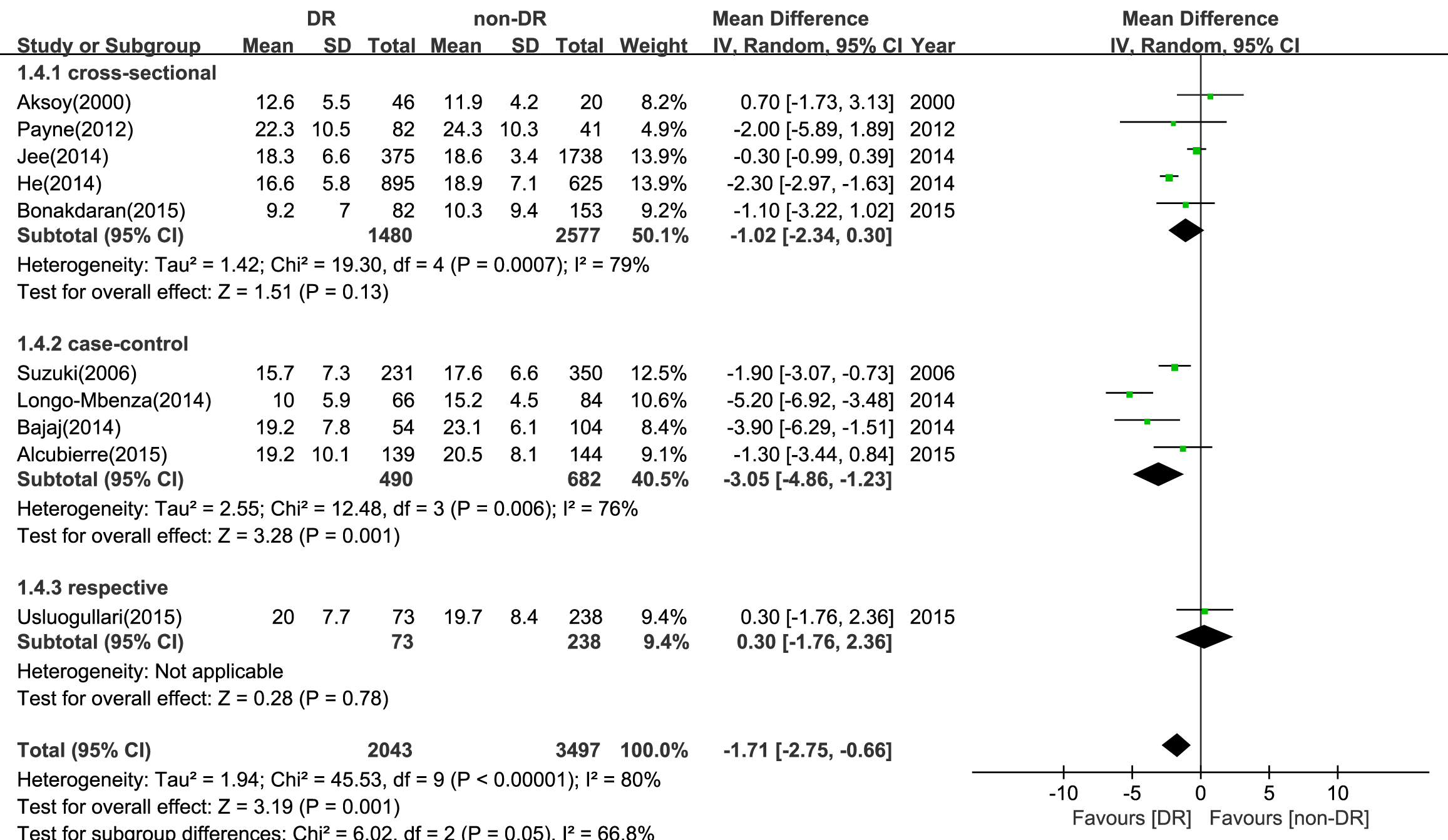


**Figure S3.** Subgroup analysis of weight mean difference (WMD) according to study design.


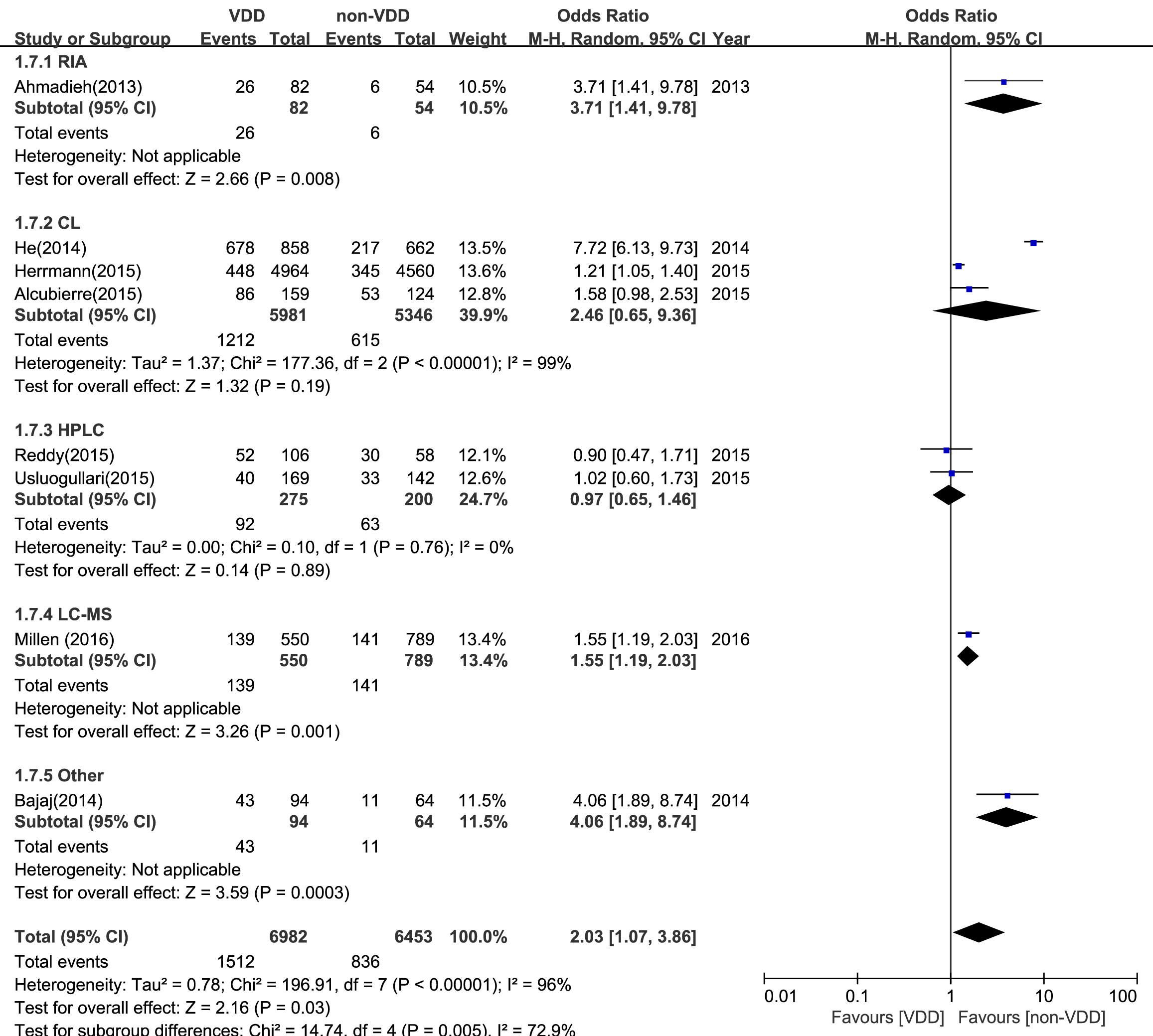


**Figure S4.** Subgroup analysis of pooled ORs according to vitamin D assay method.


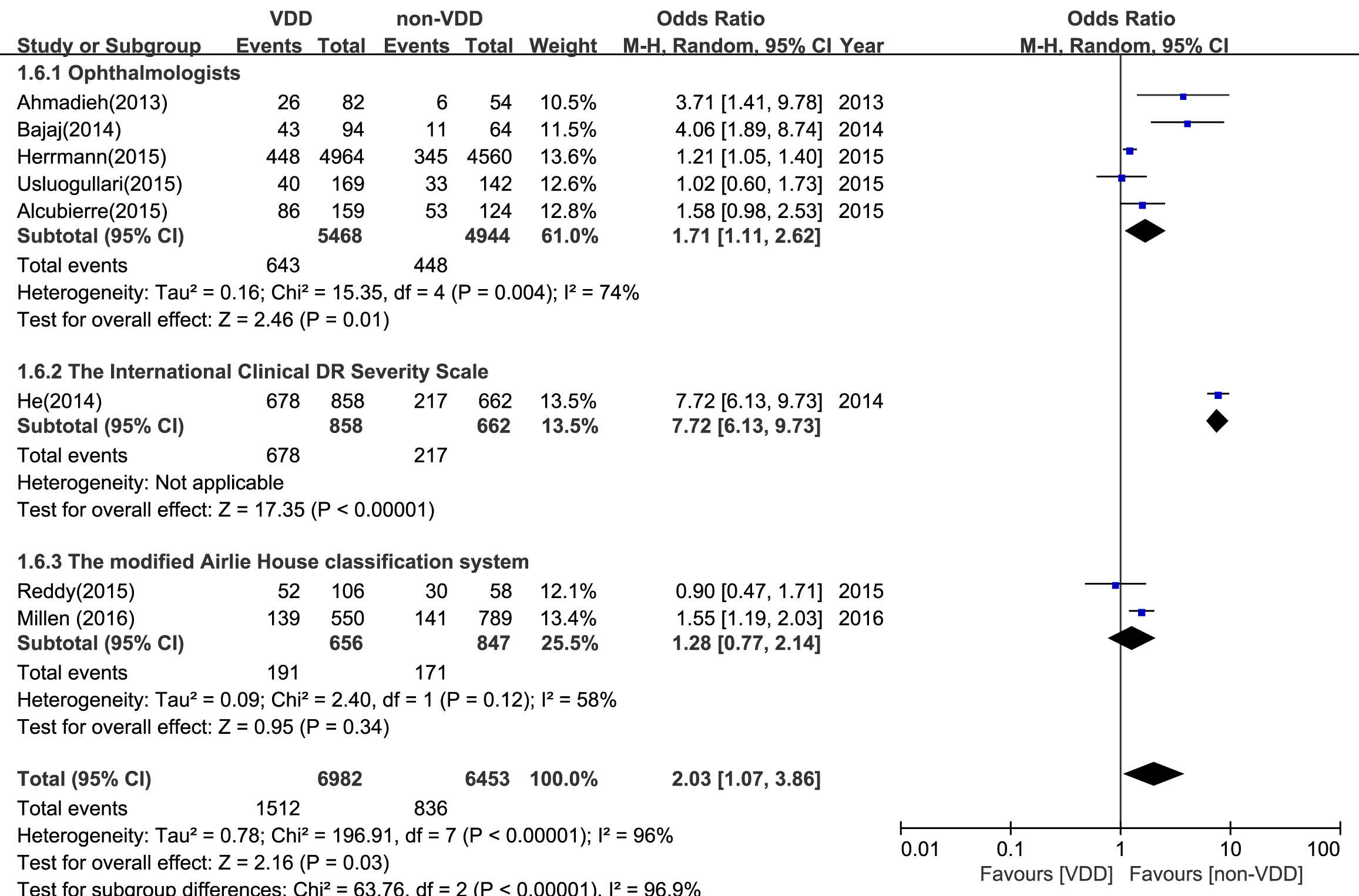


**Figure S5.** Subgroup analysis of pooled ORs according to different DR diagnosis.


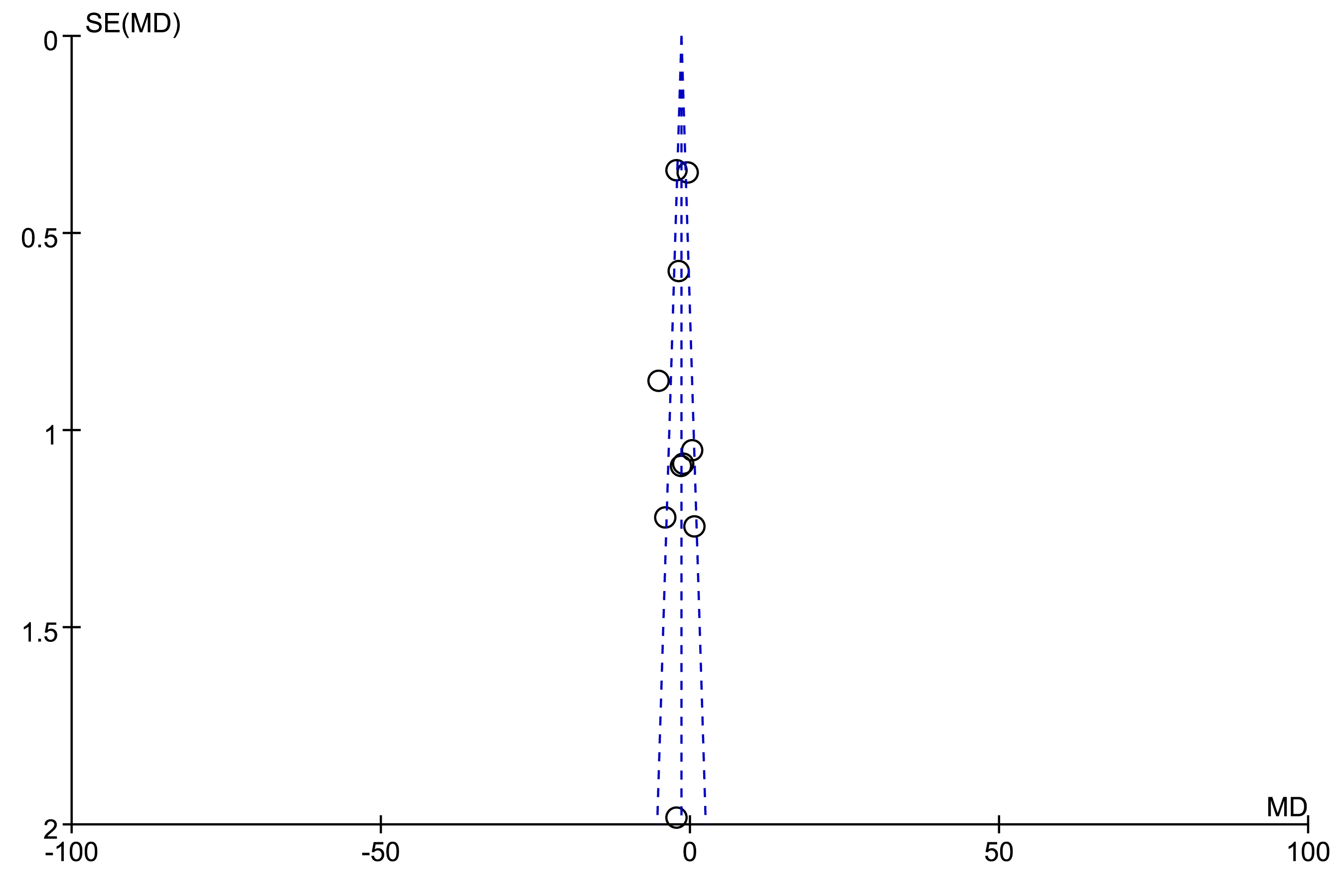


**Figure S6.** Result of funnel plots.
